# Central Venous Access Devices (CVAD) in Pediatric Oncology Patients—A Single-Center Retrospective Study Over More Than 9 Years

**DOI:** 10.3389/fped.2019.00260

**Published:** 2019-06-25

**Authors:** Olaf Beck, Oliver Muensterer, Sarah Hofmann, Heidi Rossmann, Alicia Poplawski, Jörg Faber, Jan Gödeke

**Affiliations:** ^1^Children's Hospital, Pediatric Hematology, Oncology and Hemostaseology, University Medical Center of the Johannes Gutenberg University Mainz, Mainz, Germany; ^2^Children's Hospital, Pediatric Surgery, University Medical Center of the Johannes Gutenberg University Mainz, Mainz, Germany; ^3^Institute of Clinical Chemistry and Laboratory Medicine, University Medical Center of the Johannes Gutenberg University Mainz, Mainz, Germany; ^4^Institute of Medical Biostatistics, Epidemiology and Informatics, University Medical Center of the Johannes Gutenberg University Mainz, Mainz, Germany

**Keywords:** catheter-related complications, children, vascular access devices, cancer, catheter-related infections, support care

## Abstract

**Background:** Central venous access devices (CVAD) provide important benefits in the management of oncological pediatric patients. However, these catheters are responsible for severe complications.

**Methods:** In this context, we aimed to analyze all patients receiving a CVAD in the Department of Pediatric Hematology and Oncology of the University hospital of Mainz over a period of 9 years, focused on CVAD related complications. Data on demographics, as well as intraoperative and postoperative complications were extracted.

**Results:** A total of 296 patients with a mean age 93.2 ± 62.4 months were analyzed. The majority suffered from leukemia (*n* = 91, 30.7%), lymphomas (*n* = 50, 16.9%), and brain tumors (*n* = 48, 16.2%). In 63 (21.3) patients, complications were observed. No death caused by complications of CVADs was found in our series. Catheter-related blood stream infections (BSI) (7.4%) were most prevalent, followed by dislodgements (5.4%), occlusions (2.7%), thrombosis (2.4%), and catheter leakage (2.4%). Insertion site infections were observed in three patients (1.0%). Fifty-nine percent of all patients with catheter-related BSI suffered from Leukemia. In patients with Catheter-related BSIs we detected the condition leukemia as the underlying disease as a risk factor compared to solid tumors as the underlying disease. Overall, totally implanted devices (ports) have a lower complication rate than tunneled catheter.

**Conclusion:** Implantation of CVADs seems to be safe and reliable in this large pediatric patient cohort. Even if complications occur in the long-term management of CVADs, they can be treated successfully and long-term catheter survival rates are excellent.

## Introduction

Long-term central venous access devices (CVAD) are essential in the treatment of oncological pediatric patients. These catheters are necessary tools in the application of chemotherapy, in the management of the cytostatic dilution therapy, in nutrition as well as in palliative situations. Additionally, in some pediatric patients, CVADs can be valuable for daily anesthesia application to ensure radiotherapy. Finally, frequent painful blood punctures of peripheral veins can be avoided. Therefore, CVADs improve the quality of life and the patients' safety (1). However, these catheters may lead to severe complications, e.g., infection, thrombosis and dislodgement (1, 2). Pediatric oncological patients with CVAD may represent a special entity. The management of CVAD based on local experiences and several studies emphasized a higher risk for catheter-related complications in patients with malignancies, based on the repeated cycles of chemotherapy and periods of neutropenia (3–5).

Published guidelines as well as a variety of expert groups emphasized the importance of preventing these complications (6–8). In this context, the German society for pediatric oncology and hematology (GPOH) published an evidence-base recommendation on the utilization of longterm CVADs based on a survey of the management of CVADs of 29 pediatric oncology centers (5). However, data from large pediatric cohorts receiving CVADs are scarce.

Therefore, we aimed to (i) analyze all patients receiving a CVAD in the Department of Pediatric Hematology and Oncology of the University hospital of Mainz over more than 9 years and (ii) characterize the risk of complications associated with longterm CVAD in this large pediatric oncology cohort.

## Materials and Methods

From January 2008 until April 2017, all oncologic pediatric patients and adolescents below age 18 with need of a longterm CVAD were retrospectively analyzed in the study.

Either port (Bard Access and B. Braun) CVADs, one lumen Broviac CVADs (Bard Peripheral Vascular) or two and three lumen tunneled Hickman CVADs (Bard Peripheral Vascular) were implanted. The insertion of CVAD was performed in standard sterile techniques by experienced pediatric surgeons. The size of the lumen was 5–9 Fr (French catheter gauge), depending on age and vascular status of the patient. Usually, tunneled CVAD (TCVAD) consisted of silicone (99.7%) and totally implanted catheters (ports) consisted of polyurethane (90.6%).

Data was captured from electronic and paper-based patient records and included baseline demographic information, baseline pathology, anatomical insertion site, duration of use, microbiological diagnostic and therapeutic treatment as well as complications, and side effects.

In all patients, laboratory parameters including fibrinogen, antithrombin, protein S, and protein C were controlled regularly.

Patients with thrombotic events in their history were screened for factor V Leiden [NM_000130.4: c.1601G>A, p.Arg534Gln (rs6025)], G20210A substitution in the factor II gene [NM_000506.4: c.^*^97G>A (rs1799963)], plasminogen activator-inhibitor-1 (PAI-1) gene polymorphism [NM_000602.4: c.-820_-817G(4_5) (rs587776796)], C677T [NM_005957.4: c.665C>T, p.Ala222Val (rs1801133)], and A1298C [NM_005957.4: c.1286A>C p.Glu429Ala (rs1801131)] gene polymorphism in the methylene tetrahydrofolate reductase (MTHFR).

CVAD-related complications were captured for inpatients as well as outpatients.

Complications were defined as catheter-related blood stream infection (BSI), insertion site infection, occlusion, dislodgement, thrombosis and leakage.

Catheter-related BSI was defined in patients with CVAD, clinical manifestations of infection and at least one positive blood culture obtained via CVAD. The indication to remove an infectious CVAD depended on severity and type of infection, as well as failure to clear it by targeted antibiotic therapy.

The definition of occlusion included CVADs with partial or complete blockage of the lumen. Occlusions were only taken into account in those cases in which the CVAD required replacement after it was evaluated by the pediatric surgical team. Dislodgement was defined as displacement of the tip of the CVAD into a non-central portion of the venous system.

To avoid intra-individual influences, only the first CVAD of each patient was included in this evaluation.

### Statistics

Descriptive statistics are presented as frequency, mean, or median with range as appropriate. The incidence of each complication is described using the incident rate (IR) per 1,000 catheter days and 95% confidence interval (CI). Catheter-days were the sum of follow-up from time of CVAD insertion to the CVAD removal, death or last follow-up.

Differences between groups were assessed by the chi-squared-test, Fisher's exact test or log-rank-test for categorical data. We considered the following variables as patient-related: age, gender, underlying disease; the vessel used for insertion and CVAD-related type of catheter. For univariate comparison between groups, considering the observation period, the Kaplan-Meier curve was computed and log-rank test was used.

Cox regression analysis served as univariate or multivariate model to quantify the independent contribution of one or more factors of interest on survival, expressed as the hazard ratio (HR) with 95%-CI: The local significance level was set to 5%. Multiple testing correction was not applied.

Pearson-clopper confidence intervals were analyzed by using the calculator of http://epitools.ausvet.com.au/content.php?page=CIProportion.

Statistical analyses were performed using SPSS Statistics version 23 (IBM).

### Ethics Statement

The study was approved by the local ethic committee (no: 2018-13172), and registered with German Clinical Trial Register DRKS00014944.

## Results

A total of 296 pediatric oncology patients with a mean age 93.2 ± 62.4 months entered the study. The baseline patient characteristics are displayed in Table 1.

**Table 1:** Patients characteristics, oncological diagnosis of all observed patients and patients with completed therapy.

**Mean age [months]**	**93.2** **±** **62.4**			
**Gender [male (%)]**	**149 (50.3)**			
**Underlying disease**	***n***	**%**	**Completed treatments** ***n***	**Implantation period of completed treatments in days [median (range)]**
Patients	296	100	173	337 (78–2,169)
Leukemia	91	30.7	49	326 (145–1,556)
Brain tumor	48	16.2	20	560 (265–1,133)
Lymphoma	50	16.9	41	315 (106–864)
Neuroblastoma	15	5.1	6	303 (164–902)
Soft-tissue sarcoma	17	5.7	10	321 (235–476)
Nephroblastoma	28	9.5	19	253 (83–433)
Bone malignancies	13	4.4	3	1,005 (403–1,687)
Germ cell tumor	11	3.7	10	236 (78–563)
Other solid malignancies	23	7.8	15	405 (88–2,169)

A majority of patients were suffering from leukemia (*n* = 91, 30.7%), lymphomas (*n* = 50, 16.9%), and brain tumors (*n* = 48, 16.2%).

At the end of the observation 173 (58.4%) patients completed the therapy and the catheters were removed electively. The overall indwelling implantation time of these 173 patients was in median 337 days with a range of 78–2,169 days. The respective disease entities are displayed in Table 1.

In patients suffering from leukemia and lymphomas similar implantation periods were observed with a median of 326 days and of 315 days, respectively. Patients with brain tumors and bone malignancy showed an extended implantation period of 560 and 1,005 days, respectively.

During the study period, 56 (18.9%) patients received at least one additional CVAD, prompted by tumor relapse, therapy change (e.g., indication for stem cell transplantation), and side effects. Forty-one patients received 2 CVADs, 15 patients received 3 CVADs.

Primary malposition occurred in 7 CVADs and revision was required (2.3%). All of these CVADs were tunneled. No malposition was observed in totally implanted CVADs.

### Distribution of Catheter Types and Insertion Sites

**Figure 1:**
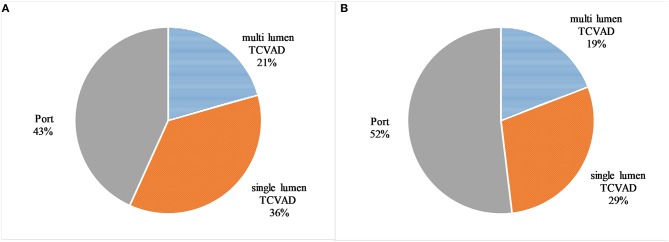
The distribution of the catheter types in the observed patients by absolute number **(A)** and the distribution of catheter types in the observed patients by catheter days **(B)**. Tunneled CVAD (TCVAD, Broviac/Hickman), and totally implanted CVAD (Port).

Tunneled CAVDs (TCVADs) were placed in 168 patients (56.8%), while 128 patients received totally implanted CVADs (43.2%). Single-lumen Broviac catheters were placed in 107 patients (36.2%), while 61 of the TCVADs catheters were multi-lumen Hickman catheters (20.6%) (Figure 1A).

The jugular vein was used in 151 (51.0%) for insertion, while 145 (49.0%) catheters were inserted into the subclavian or cephalic vein. In 260 (87.8%) cases, the catheter was implanted on the right side of the chest and only in 36 (12.2%) cases the catheter was implanted on the left side.

### Catheter Days (CD)

In total 99,633 catheter days (CD) were recorded in 296 patients with a median of 284.5 CD (range 1–2,169). Overall, 47,921 (48.1%) CD were documented in TCVADs, while 51,712 (51.9%) CD were observed in totally implanted CVADs (Figure 1B).

### Complications

**Figure 2:**
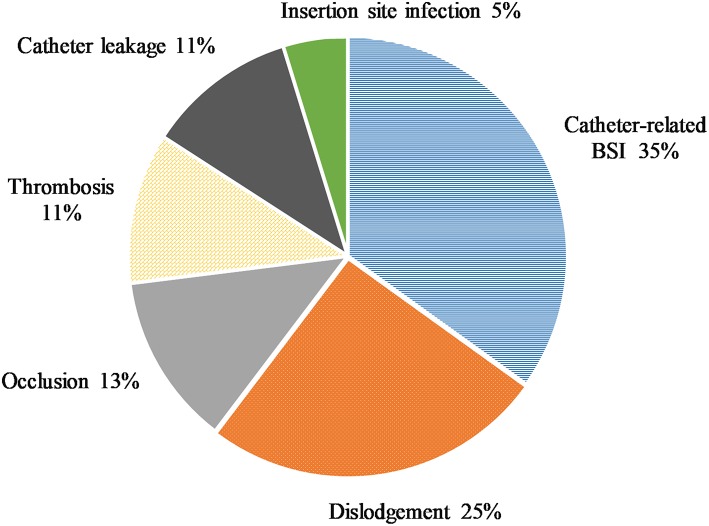
The distribution of complications in the observed patients.

In 63 (21.3) patients, complications were observed (Table 2 and Figure 2). No deaths caused by complications of CVAD were recorded in our series over a period of 9.3 years.

**Table 2:** Long-and short-term complication of all observed patients.

**Long-and short-term-complication**	**Number of complication *n* (%)**	**Incidence rate (per 1,000 catheter days) and 95%-CI**
Complications overall	63 (21.3)	0.63 (0.49–0.81)
Catheter-related BSI	22 (7.4)	0.21 (0.14–0.33)
Dislodgement	16 (5.4)	0.16 (0.09–0.26)
Occlusion	8 (2.7)	0.08 (0.04–0.15)
Thrombosis	7 (2.4)	0.07 (0.03–0.15)
Catheter leakage	7 (2.4)	0.07 (0.03–0.15)
Insertion site infection	3 (1.0)	0.03 (0.01–0.09)

*N, number of complications and percentage of whole observed group*.

Catheter-related blood stream infections (BSI) (*n* = 22, 7.4%) were most prevalent, followed by dislodgements (*n* = 16, 5.4%) with an incidence rate of 0.16.

Less frequent complications were occlusions (*n* = 8, 2.7%), thrombosis (*n* = 7, 2.4%), and catheter leakage (*n* = 7, 2.4%). Insertion site infections were observed in three patients (1.0%). In no patient catheter associated cardiac arrhythmia was detected.

To compare the rate of complications with previous studies, incidence rates of complications (per 1,000 catheter days) was displayed in Table 2.

#### Catheter-Related Bloodstream Infection (BSI)

In 22 (7.4%) patients, who had clinical signs of infection, a positive blood culture was detected and a diagnosis of catheter-related BSI was thereby established. In four of these patients, two pathogens were found.

Beside gram negative agents like *Escherichia coli* (*n* = 7) and *Pseudomonas* (*n* = 2), gram positive organisms as *Staphylococcus aureus* (*n* = 2), coagulase (–) negative staphylococcus (*n* = 4) and *Streptococcus mitis* (*n* = 3) could be identified repeatedly. Four pathogens were found only in one episode. The results of all positive blood cultures are listed in Table 3.

**Table 3:** Microbiological data of 22 episodes of possible catheter-related bloodstream infections (BSI) in the observed patient group.

**Patient**	**Age in years**	**Disease**	**CVAD**	**BSI on day**	**Agent**	**Treatment**
1	16	ALL	TCVAD	50	*Escherichia coli*	Conservative
2	7	AML	TCVAD	79	*Streptococcus mitis*	Conservative
3	3	ALL	TCVAD	43	*Haemophilus influenzae*	Conservative
4	17	Lymphoma	Port	39	*Staphylococcus aureus*	Conservative
5	16	Lymphoma	Port	23	*Staphylococcus epidermidis Staphylococcus haemolyticus*	Conservative
6	10	Lymphoma	TCVAD	76	*Enterococcus faecalis Escherichia coli*	Conservative
7	16	Germ cell tumor	TCVAD	72	*Staphylococcus epidermidis*	Conservative
8	11	ALL	TCVAD	42	*Escherichia coli*	Conservative
9	0	Neuroblastoma	TCVAD	428	*Staphylococcus aureus*	Removal
10	14	AML	TCVAD	106	*Granulicatella adiacens*	Conservative
11	3	AML	TCVAD	89	*Streptococcus mitis*	Conservative
12	6	ALL	TCVAD	229	*Escherichia coli*	Removal
13	6	Brain tumor	TCVAD	268	*Bacillus cereus*	Removal
14	5	ALL	TCVAD	154	*Escherichia coli*	Removal
15	4	Other malignancy	TCVAD	478	*Pseudomonas aeruginosa*	Removal
16	15	ALL	Port	139	*Escherichia coli Klebsiella pneumoniae*	Removal
17	9	AML	Port	600	*Escherichia coli*	Removal
18	16	Lymphoma	Port	261	*Staphylococcus epidermidis*	Conservative
29	9	ALL	Port	762	*Bacteroides fragilis*	Conservative
20	8	ALL	Port	221	*Streptococcus mitis Streptococcus salivarius*	Conservative
21	5	ALL	Port	51	*Pseudomonas aeruginosa*	Conservative
22	11	Brain tumor	Port	1,173	*Serratia liquefaciens*	Conservative

*Tunneled CVAD (TCVAD, Broviac/Hickman); totally implanted CVAD (Port)*.

The detection of *E. coli* was more frequent in patients with TCVAD (5/13; 38.5%) in comparison to patients with totally implanted CVADs (2/9; 22.2%).

Of all patients with catheter-related BSI, 59.1% suffered from a primary diagnosis of Leukemia (*n* = 13).

**Figure 3:**
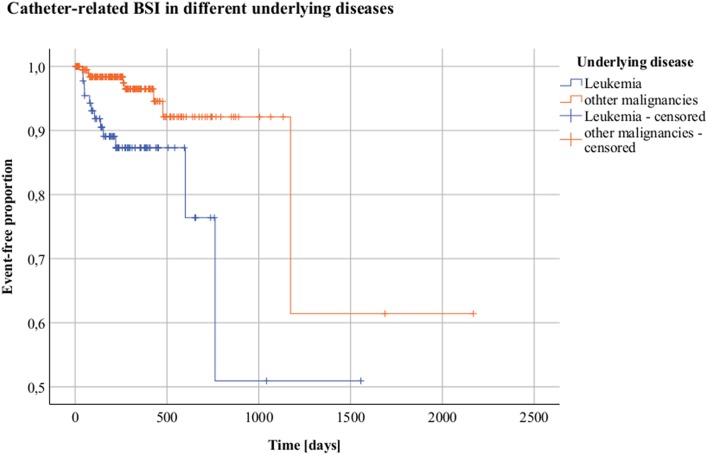
Catheter-related bloodstream infections (BSI) in different underlying diseases. Kaplan-Meier analysis Y = Event-free proportion. X = Time [days]. Orange = Other malignancies; blue = Leukemia.

Leukemia was found to be a risk factor for catheter-related BSIs, compared to solid malignancies as the underlying disease (*p* = 0.004) in univariate survival analysis with an HR of 3.734 (95%-CI: 1.520–9.170). The time interval from implantation to BSI detection was also 38% shorter for patients with leukemia (Figure 3).

Primary antibiotic treatment was successful in 15 (68.2%) patients, revision was required in 7 (31.8%) patients.

#### Dislodgement

Dislodgement of the catheter tip was detected in 16 patients, with the catheter tip no longer in a central vein. The risk of dislodgement was higher with TCVAD compared with totally implanted catheters with an HR of 6.218 (95%-CI: 1.412–27.392; *p* = 0.016). It was also negatively associated in patients with younger age with an HR of 0.828 (95%-CI: 0.722–0.949; *p* = 0.007) per year of live. As the type of the CVAD and age of the patient were found to be highly correlated, these factors were not included into the multivariate analysis.

#### Insertion Site Infection

Insertion site infection of CVADs was reported in three patients with the proof of *Pseudomonas aeruginosa* (*n* = 1) and *S. aureus* (*n* = 2). Insertion site infection occurred only in patients with TCVAD (Table 4).

**Table 4:** Microbiological data of three episodes of local infections in the observed patient group.

**Patient**	**Age in years**	**Disease**	**CVAD**	**Infection on day**	**Agent**	**Treatment**
1	6	Soft tissue sarcoma	TCVAD	429	*Pseudomonas aeruginosa*	Conservative
2	2	Brain tumor	TCVAD	44	*Staphylococcus aureus*	Removal
3	1	Neuroblastoma	TCVAD	740	*Staphylococcus aureus*	Removal

*Tunneled CVAD (TCVAD, Broviac/Hickman)*.

#### Thrombotic Complications

Seven patients with thrombotic complications were identified. In three of these patients, upper extremity thrombosis (subclavian and/or axillary vein) was found. Four of seven suffered from pulmonary embolism (PE) including one patient with peripheral PE.

Under systemic thrombolytic therapy (Actilyse; Alteplase rtpa) or anticoagulation therapy, all thrombotic events were treated successfully, all patients survived and continued the chemotherapy.

All patients with PE were diagnosed in the first 50 days after implantation, and in 3 of 4 patients, a multi-lumen TCVAD was implanted.

Five of seven patients with thrombotic event were older than 14 years.

Genetic analyses revealed one heterozygous prothrombin G20210A mutation in a patient with PE. In no patient, a higher thrombophilia risk associated with factor V Leiden could be found. However, in 5 of 6 patients, at least one mutation in the PAI-1 (4G/5G), and in all analyzed patients (*n* = 5) at least one mutation in the MTHFR (C677T) or (A1298C) were detected. The patient without PAI-1 mutation suffered from obesity (BMI > 30).

The affected patients are listed in Table 5.

**Table 5:** Patients with thrombotic events in the observed patient group.

**Patient**	**Age**	**BMI**	**Disease**	**CVAD and location**	**Thrombotic event on day**	**Specified thrombosis/location**	**Genetic predisposition**
1	14	30.7	Lymphoma	Port/Cephalic vein	22	Thrombosis in axillary vein	Homozygous PAI-mutation 4G/4G, Heterozygous MTHFR A1298C mutation
2	16	25.2	ALL	Port/Cephalic vein	10	Pulmonary embolism	Heterozygous II G20210A mutation, Heterozygous PAI-mutation 4G/5G, Heterozygous MTHFR A1298C mutation
3	16	24.9	ALL	TCVAD/Internal jugular vein	50	Pulmonary embolism	Negative (II and V), others not done
4	7	13.7	ALL	TCVAD/Cephalic vein	221	Thrombosis in subclavian vein	Heterozygous PAI-mutation 4G/5G, Heterozygous MTHFR C677T mutation
5	16	30	Lymphoma	Port/Subclavian vein	13	Thrombosis in axillary and subclavian vein	Negative (II and V and PAI), others not done
6	4	14.8	ALL	TCVAD/Internal jugular vein	9	Pulmonary embolism	heterozygous PAI-mutation 4G/5G, Heterozygous MTHFR C677T mutation
7	15	37.7	Bone cancer	TCVAD/Internal jugular vein	6	Pulmonary embolism	Heterozygous PAI-Mutation 4G/5G, Heterozygous MTHFR A1298C mutation

*Tunneled CVAD (TCVAD, Broviac/Hickman); totally implanted CVAD (Port)*.

#### Occlusion

Six of eight CVADs with occlusion were removed and replaced. Two of eight were successfully re-opened after repeated applications of urokinase.

### Differences in Complications

#### Differences in Complications of Tunneled vs. Totally Implanted CVADs

Complication rates of tunneled vs. totally implanted CVADs were compared.

Overall, total number of complications events was low. However, in all analyzed complication categories the IR was higher in TCVAD (Table 6) compared to totally implanted (port) devices. The highest difference was found concerning dislodgement.

**Table 6:** Incidence of complications (per 1,000 catheter days) of tunneled CVAD (TCVAD, Broviac/Hickman) and totally implanted CVAD.

	**Incidence rate (per 1,000 catheters days)**		**Rate ratio**
**Complication**	**TCVADs**	**Ports**	**TCVAD vs. port**
Complications overall	0.835 (0.596–1.136)	0.309 (0.177–0.502)	2.702
Catheter-related BSI	0.271 (0.144–0.464)	0.174 (0.08–0.330)	1.557
Dislodgement	0.292 (0.160–0.490)	0.039 (0,005–0.140)	7.487
Occlusion	0.125 (0.046–0.273)	0.039 (0,005–0.140)	3.205
Thrombosis	0.083 (0.023–0.214)	0.058 (0.012–0.170)	1.431

The multivariate analysis of the risk of catheter associated complications per type of CVAD showed a higher risk for TCVADs than for totally implanted catheters with a HR of 2.893 (95%-CI: 1.332–6.186; *p* = 0.007).

Dividing TCVADs into single- and multi-lumen TCVADs, the analysis was repeated. The HR for complications in patients with multi-lumen TCVADs compared to totally implanted catheters was 3.421 (95%-CI: 1.712–6.835; *p* > 0.001) and 2.583 (95%-CI: 1.340–4.979; *p* = 0.005) for single-lumen TCVADs compared to totally implanted catheters, respectively. No significant association was found between single-lumen TCVADs and multi-lumen TCVADs.

**Figure 4:**
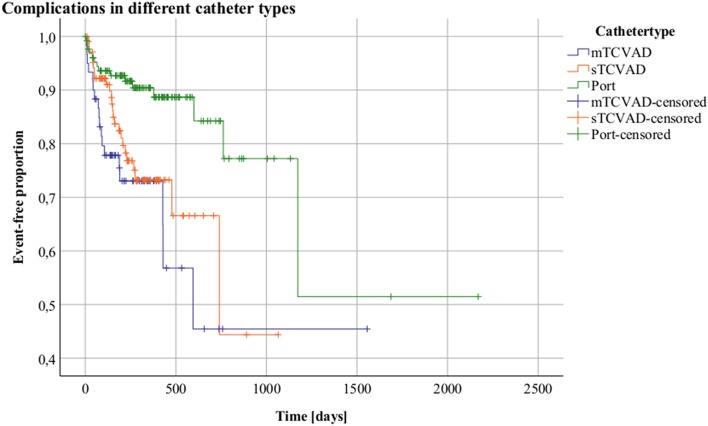
Complications in different catheter types. Kaplan-Meier analysis Y = Event-free proportion. X = Time [days]. Tunneled CVAD: (TCVAD, Broviac/Hickman); totally implanted CVAD: (Port). Blue = multi-lumen TCVAD, orange = single-lumen and green = totally implanted CVAD.

The distribution of complications in relation to catheters types is presented in Figure 4.

However, in BSI, the catheter type was not found to affect the risk of catheter-related BSI, but univariate analysis showed a higher risk for catheters with more than one lumen compared to all kind of single-lumen catheters with an HR of 3.465 (95%-CI: 1.380–8.700; *p* = 0.008).

#### Complication Under Consideration of the Primary Diagnosis

As described, a higher risk for patients suffering from leukemia than for patients suffering from solid malignancies in terms of catheter-related BSI (as a complication) was found.

#### Complications and Gender

No gender specific differences could be detected in our series.

### Status of CVAD at the Endpoint of the Observation Period

At the endpoint of the observation period, 173 out of 296 (58.4%) CVADs were removed electively after the end of the therapy, 33 (11.1%) CVADs had been explanted due to complications and 54 (18.2%) CVADs were still *in situ*. In this study, 20 (6.5%) patients died because of the underlying disease. In 16 (5.4%), other reasons such as CVAD change as a consequence of a therapy change (*n* = 12) or an accidental removal (*n* = 4) lead to the end of the observation period.

## Discussion

In the present large retrospective study, we report about pediatric oncology patients with CVADs over a period of more than 9 years. We recorded patient and implanted catheter characteristics and complications in 99,633 catheter days in 296 patients, making this one of the largest single-center studies on pediatric CVADs for oncologic indications.

The number of implanted Port catheters seemed to be high in comparison to other oncology units (5). Even in pediatric patients with non-Hodgkin lymphoma and leukemia totally implanted CVADs were preferred, as long as a stem cell transplantation was not expected in the future. Furthermore, the treatment protocols of solid tumors, e.g., in brain tumors and soft-tissue sarcomas permit the implantation of totally implanted CVADs.

In order to avoid regular antiseptic cleaning at the entry site of TCVADs, adolescent patients, who organize the personal hygiene by themselves, prefer totally implanted CVADs. The option to hide a totally implanted CVAD under the skin may be another reason making these devices attractive for this patient group. Several recommendations pointed out the possibility of early removal of TCVADs after the completion of therapy (5). In our cohort, totally implanted CVADs were longer accepted by patients even after the end of the therapy (Table 1).

In comparison to previous analyses, the overall risk for complications associated with longterm CVAD in this large pediatric oncology cohort was similar.

The most frequent complications in our pediatric patients were catheter-related BSIs which were increased in patients with leukemia in comparison to patients with other malignancies. Catheter-associated complications were lower for totally implanted CVADs than for TCVAD. Particularly, the ratio of occlusions in TCVADs was 3-fold higher than in totally implanted CVADs.

Most importantly, no CVAD associated death was found in our series over a period of 9 years.

### BSI in CVAD

BSI and fever of unknown origin frequently occur in oncological patients. A positive blood culture identifies the pathogen in a relatively low number of patients only (9–11). In many cases, a definitive diagnosis would require catheter removal and microbiological analysis of the catheter tip.

In a metaanalysis of 74 studies in pediatric patients, including oncological as well as benign indications for CVAD implantation, BSI associated complication rate of CVAD was specified with a pooled rate (*n* = 50 studies) of 1.63 per 1,000 catheter days (95%-CI: 1.40–1.86) (2). Further authors published rates of 0.46–1.40 per 1,000 catheter days and 0.1–2.3 per 1,000 catheter days, respectively (12–14).

In this study, more than 39.0% of all complications were due to infectious agents with an IR of 0.25 per 1,000 catheter days (95%-CI: 0.16–0.37), predominately in patients with leukemia (15).

Our low IR could be explained by the high number of totally implanted CVADs, as previous studies revealed a significant higher rate of BSI in tunneled than in totally implanted CVADs (15–17).

Interpretation of the above mentioned results may be complicated as the classification criteria for catheter–related BSI or for insertion site infection varies between studies. Based on non-randomized studies, the preference of single-lumen over multi-lumen catheters has been recommended. In contrast to our results in children, randomized studies in adults have shown no correlation between infection rates and the number of lumina (8, 18, 19).

### Blood Culture Results

Gram positive bacteria, in particular coagulase-negative staphylococci, *S. mitis* and *St. aureus* are the most commonly found pathogens in blood cultures of oncological patients with catheter-related infections (8). This could be confirmed in our patient population. Additionally, gram negative agents, e.g., *E. coli* and *Pseudomonas* were found. Surprisingly no fungus infection could be detected. The higher frequency of *E. coli* in TCVADs could be caused by smear infections.

In clinical practice, the majority of BSI can successfully be managed by antibiotics without removing the CVAD (15, 20–22). In our cohort only 31.8% of the contaminated CVADs were removed, similar to Newman et al. (29.2%).

### Insertion Site Infection

Only three severe local CVAD infections occurred in our cohort. In 2 patients, the catheter was removed. This comparably low local infection rate may be prompted by a well-established local standard containing regular (every other day) antiseptic cleaning by using octenidin/phenoxyethanol, a sterile gauze and tape dressing at the entry site of the catheter as published before (5). Before the first discharge from hospital after CVAD implantation, we make sure that parents or caregivers have to demonstrate the ability to perform the catheter handling under maximal sterile precaution.

### Thrombotic Complications

Thrombotic events in pediatric patients with CVADs are rare and routine thromboprophylaxis does not seem to reduce the risk of thrombosis (23, 24).

In comparison to Revel-Vilk al. (IR 0.13 per 1,000 catheter days) and to the pooled data of Ullman et al. (2) the risk of thrombosis is similar in our cohort.

In all patients but one, who suffered from obesity, at least one PAI-1 mutation and in all analyzed patients a MTHFR mutation was detected. In contrast, thrombophilic markers were not associated with CVAD-related thrombosis or occlusion in previous studies in the consideration of factor V Leiden, G20210A substitution in the factor II gene and C677T in MTHFR (1). However, the mutation of PAI-1 and the mutation of A1298C in MTHFR were not included. In our cohort, not all patients were investigated for possible thrombophilia. However, the detected mutation of PAI-1 in combination with the mutation of MTHFR could possibly be associated with an increased risk of thrombotic events and deserves further evaluation.

Apart from one patient suffering from osteosarcoma, all other patients were suffering from Leukemia and Lymphoma. In all these disease entities, high dose methotrexate is an essential component of the treatment protocol. Besides evaluation of thrombophilic markers, the measurement of homocysteine could be a possible tool to specify the risk of thrombotic complications under chemotherapy in these patients (25).

### Dislodgement

Especially in pediatric patients, dislodgement of the CVAD is a common complication and is usually defined as any event causing the catheter tip to migrate from the superior vena cava. The pooled data of Ullman et al. show a significantly higher rate of dislodgement per 1,000 catheter days for TCVADS with an IR of 0.24 (95%-CI: 0.03–0.46) compared to totally implanted catheters with an IR of 0.02 (95%-CI: 0.00–0.04). These incident rates are in line with our overall IR for dislodgements of 0.16 (95%-CI: 0.09–0.26).

### Occlusion

Occlusions of CVADs can be caused by the presence of a fibrin sheath, a catheter tip thrombus or if the catheter tip is being positioned against the vessel wall (26). A positive family history of thrombosis has been shown to significantly increase the risk of occlusions (1). In previously published studies, occlusion occurred at an IR of 2.0–2.8 per 1,000 catheter days and an IR of 1.35 per 1,000 catheter days (95%-CI: 1.1–1.63), respectively (1, 27–29).

Pooled studies in pediatric patients revealed a pooled IR of 0.85 per 1,000 catheter days (95%-CI: 0.48–1.23) in TCVADs (*n* = 7 studies), and a pooled IR of 0.3 per 1,000 catheter days (95%-CI: 0.04–0.57) in totally implanted CVADs (*n* = 5 studies). These incident rates are considerably higher than in our study 0.08 (95%-CI: 0.04–0.15). However, in contrast to other studies, only CVADs were included, where usual re-open procedures could not be performed.

Notably, the rate of occlussions in TCVADs was 3-fold higher than in totally implanted CVADs. This confirms the results of previous examinations (2).

As recently published, catheter-resistance monitoring maybe a helpful tool to predict catheter occlusion in advance (26).

### Cardiac Tamponade

Catheter–related cardiac perforation with subsequent tamponade is a highly lethal complication with a mortality in adults up 65–78% (30–32). This is a very rare event. No catheter-related tamponade, no case of cardiac perforation and no catheter-related arrhythmia was observed in our cohort.

### Catheter Days

The metaanalysis of 74 cohort studies by Ullman et al. in pediatric patients with several not only oncological indications for CVAD implantation revealed that 25% of CVADs failed before completion of therapy at a rate of 1.97 per 1,000 catheter days (95%-CI: 1.71–2.23) (2). Subgroup analyses in oncolocigal pediatric patients showed a pooled IR of 0.88 per 1,000 catheter days (95%-CI: 0.41–1.34) in TCVADs (*n* = 5 studies), and a pooled IR of 0.15 per 1,000 catheter days (95%-CI: 0.09–0.20)/1,000 in totally implanted CVADs (*n* = 10 studies). These rates are comparable to the overall IR of 0.63 per 1,000 catheter days (95%-CI: 0.49–0.81), as well as the IR of 0.84 per 1,000 catheter days (95%-CI: 0.60–1.14) in TCVAD and the IR of 0.31 per 1,000 catheter days (95%-CI: 0.18–0.50) in totally implanted CVADs in our cohort.

Limitations of our study include the retrospective design as well as the single-center setup. The management of CVAD is based on local experiences and our study only reflects outcome measures in this specific pediatric patient cohort.

In patients with malignant diseases, a prolonged hospital stay should be avoided. In this context, Gaur et al. published in 2017 a prospective, multi-center study on the prevalence of BSI in pediatric oncology patients. Within 2 years, 34 centers registered 1110 BSIs and revealed across-center differences (33). Such a prospective registry would be valuable for recording of other CVAD-associated complications in order to detect prevalence rates and cross-center differences.

## Conclusion

Although implantation of CVADs seems to be safe and reliable in this large pediatric patient cohort, we draw several conclusions from our findings. Catheter-related BSIs and dislodgement are the most frequent complications, followed by occlusion and thrombosis. A primary diagnosis of leukemia is a risk factor for infection. Overall, totally implanted CVADs have a lower complication rate than tunneled catheters, particularly regarding a much lower risk of dislodgement. Even if complications occur in the long-term management of CVADs, they can usually be treated successfully. Long-term catheter survival rates are excellent and mortality from catheter-related causes is extremely low in pediatric oncologic patients.

## Ethics Statement

The study is a retrospective study and elevates only clinical data. The study was approved by the local ethic committee (no: 2018-13172), and registered with German Clinical Trial Register DRKS00014944. Local ethic committee: Landesärztekammer Rheinland-Pfalz, Körperschaft des öffentlichen Rechts, Deutschhausplatz 3, 55116 Mainz.

## Author Contributions

OB, JG, SH, HR, JF, and OM contributed to the conception and design of this study. SH and OB collected data, drafted, and wrote the manuscript. SH and AP performed the statistical analysis. HR performed laboratory analysis. HR, JF, JG, AP, and OM critically reviewed the manuscript. All authors read and approved the final manuscript.

### Conflict of Interest Statement

The authors declare that the research was conducted in the absence of any commercial or financial relationships that could be construed as a potential conflict of interest.

## Abbreviations

CVAD: Central venous access devices
GPOH: German society for pediatric oncology and hematology
Fr: French catheter gauge
PAI-1: plasminogen activator-inhibitor-1
MTHFR: methylene tetrahydrofolate reductase
BSI: blood stream infection
IR: incident rate
CI: confidence interval
HR: hazard ratio
IMBEI: Institute of Medical Biostatistics Epidemiology and Informatics (IMBEI)
SD: standard deviation
CD: catheter days
PE: pulmonary embolism
TCVAD: tunneled central venous access devices
BMI: Body mass index
Fig: Figure
Tab: Table.
